# Supplementary material for: Dietary Inflammatory Index, Obesity, and the Incidence of Colorectal Cancer: Findings from a Hospital-Based Case-Control Study in Malaysia
Source: Nutrients. 2023 Feb 16;15(4):982. doi: 10.3390/nu15040982 (PMC9965675; doi:10.3390/nu15040982)
Supplement: Supplementary file 1 [file nutrients-15-00982-s001.zip › nutrients-2152440-supplementary.pdf]

Supplementary Materials: Dietary Inflammatory Index, Obesity, and Incidence of Colorectal Cancer: Findings from Hospital-Based Case-Control Study in Malaysia

Nor Hamizah Shafiee[1], Nurul Huda Razalli[2,3,4], Mohd Razif Shahril,[4] Khairul Najmi Muhammad Nawawi[3,5], Norfilza Mohd Mokhtar[3,6], Ainaa Almardhiyah Abd Rashid[7], Lydiatul Shima Ashari[7], Hamid Jan Jan Mohamed[7], and Raja Affendi Raja Ali[3,8,*]

Table S1. Intakes of 29 food parameters according to quartile of the energy-adjusted dietary inflammatory index (E-DII) score and BMI categories.

Food Parameters	BMI < 25 kg/m² n = 157					BMI ≥ 25 kg/m² n = 156				
	Quartiles of E-DII					Quartiles of E-DII				
	Q1	Q2	Q3	Q4	*p*-Value [a]	Q1	Q2	Q3	Q4	*p*-Value [a]
Carbohydrate (g/day)	244.82 ± 22.5	254.49 ± 22.0	256.87 ± 28.1	275.16 ± 31.9	< 0.001	301.25 ± 20.1	313.41 ± 46.7	323.34 ± 24.1	367.89 ± 38.3	< 0.001
Protein (g/day)	66.33 ± 8.3	84.67 ± 7.3	85.67 ± 8.4	93.98 ± 8.5	< 0.001	86.25 ± 10.5	80.67 ± 13.0	102.00 ± 12.0	115.01 ± 17.0	< 0.001
Total fat (g/day)	51.77 ± 7.8	58.70 ± 6.7	63.47 ± 12.1	73.15 ± 10.8	< 0.001	71.05 ± 9.4	79.64 ± 11.4	84.58 ± 12.9	99.15 ± 18.0	< 0.001
Fibre (g/day)	22.04 ± 5.8	16.14 ± 3.7	13.24 ± 2.7	10.01 ± 3.0	< 0.001	19.31 ± 6.2	17.24 ± 3.6	14.02 ± 3.6	8.55 ± 3.0	< 0.001
ω-3 Fatty acids (g/day)	1.48 ± 0.4	1.26 ± 0.5	1.21 ± 0.4	0.73 ± 0.4	< 0.001	1.19 ± 0.8	1.21 ± 0.4	0.85 ± 0.7	0.50 ± 0.3	< 0.001
ω-6 Fatty acids (g/day)	2.24 ± 1.9	2.65 ± 2.0	1.89 ± 0.9	1.68 ± 0.9	0.085	3.67 ± 2.4	3.48 ± 2.1	3.30 ± 2.2	2.08 ± 0.6	0.530
SFA (g/day)	26.01 ± 2.5	28.64 ± 2.3	30.78 ± 4.1	37.68 ± 3.1	< 0.001	32.33 ± 2.3	39.36 ± 4.6	43.36 ± 3.7	50.76 ± 4.7	< 0.001
Trans fat (g/day)	0.71 ± 0.6	0.51 ± 0.5	0.74 ± 0.5	0.99 ± 0.7	0.071	1.16 ± 0.7	1.12 ± 0.7	1.29 ± 0.8	1.92 ± 0.7	< 0.001
MUFA (g/day)	19.44 ± 3.8	25.18 ± 2.8	28.65 ± 2.1	32.08 ± 2.7	0.222	31.84 ± 4.7	35.00 ± 3.8	36.71 ± 4.2	44.30 ± 2.2	0.106
PUFA (g/day)	5.61 ± 3.5	4.37 ± 3.0	3.30 ± 1.3	2.40 ± 3.6	< 0.001	5.72 ± 3.8	4.16 ± 2.4	3.22 ± 3.7	2.18 ± 2.7	< 0.001
Cholesterol (mg/day)	447.88 ± 36.5	362.51 ± 125.0	372.53 ± 101.2	396.98 ± 69.8	0.088	445.91 ± 136.2	502.84 ± 110.3	425.12 ± 132.2	528.79 ± 68.9	0.201
Thiamin (mg/day)	1.07 ± 0.2	0.93 ± 0.3	0.78 ± 0.2	0.77 ± 0.3	< 0.001	1.01 ± 0.3	0.90 ± 0.2	0.96 ± 0.3	0.62 ± 0.2	< 0.001
Riboflavin (mg/day)	1.83 ± 0.3	1.54 ± 0.3	1.47 ± 0.4	1.23 ± 0.4	< 0.001	1.73 ± 0.5	1.63 ± 0.3	1.45 ± 0.4	1.07 ± 0.4	< 0.001
Niacin (mg/day)	14.93 ± 2.8	13.61 ± 4.3	11.57 ± 1.7	11.49 ± 9.3	0.003	14.51 ± 4.6	12.47 ± 2.1	11.10 ± 1.9	9.56 ± 4.2	< 0.001
Folic acid (ug/day)	282.04 ± 64.9	271.46 ± 56.5	232.54 ± 55.8	221.61 ± 57.8	0.004**	231.08 ± 71.8	246.21 ± 60.8	257.56 ± 81.6	199.11 ± 54.7	0.070
Vitamin B6 (mg/day)	2.56 ± 1.8	2.08 ± 0.4	1.53 ± 0.3	1.20 ± 0.7	< 0.001	3.11 ± 0.3	1.89 ± 0.1	1.83 ± 1.2	1.02 ± 0.4	< 0.001
Vitamin B12 (mg/day)	5.95 ± 0.7	4.79 ± 1.5	5.01 ± 1.4	4.95 ± 1.2	0.116	3.63 ± 1.4	1.89 ± 1.2	1.83 ± 1.5	1.02 ± 1.0	0.173
Vitamin A (RE/day)	1518.37 ± 405.7	1478.73 ± 298.9	1273.54 ± 246.2	1468.35 ± 283.7	0.186	1450.05 ± 241.9	1216.74 ± 249.4	1269.81 ± 265.7	1121.15 ± 306.3	0.011**
Vitamin C (mg/day)	176.09 ± 45.4	118.42 ± 21.8	113.42 ± 44.6	60.39 ± 32.1	< 0.001	168.20 ± 89.2	140.52 ± 21.5	79.11 ± 45.3	44.04 ± 38.8	< 0.001

Vitamin D (ug/day)	4.74 ± 0.4	5.02 ± 1.0	4.34 ± 1.0	4.49 ± 0.8	0.061	5.15 ± 1.1	4.79 ± 0.5	5.19 ± 0.8	4.99 ± 0.7	0.545
Vitamin E (mg/day)	10.38 ± 2.4	7.85 ± 0.7	7.56 ± 0.7	6.43 ± 0.7	< 0.001	15.35 ± 1.1	8.02 ± 0.8	7.25 ± 1.7	4.42 ± 1.6	< 0.001
ß-Carotene (ug/day)	3456.34 ± 1048.7	2103.41 ± 259.4	1988.34 ± 250.6	1246.30 ± 423.1	< 0.001	2648.91 ± 691.3	2456.97 ± 417.8	1426.50 ± 336.5	914.8 ± 460.0	< 0.001
Iron (mg/day)	14.83 ± 2.8	16.58 ± 2.7	18.60 ± 2.0	20.90 ± 3.1	< 0.001	15.45 ± 2.2	16.59 ± 1.5	17.17 ± 1.6	20.89 ± 2.2	< 0.001
Magnesium (mg/day)	361.62 ± 36.4	344.01 ± 19.3	255.32 ± 20.7	201.71 ± 31.6	< 0.001	524.09 ± 41.3	296.43 ± 21.0	271.80 ± 22.0	182.13 ± 16.3	< 0.001
Selenium (ug/day)	156.19 ± 22.7	162.43 ± 15.2	143.41 ± 27.1	159.60 ± 21.7	0.126	140.61 ± 15.5	120.30 ± 22.6	123.58 ± 18.4	115.55 ± 13.3	0.004**
Zinc (mg/day)	15.19 ± 2.9	9.75 ± 1.0	7.16 ± 0.7	6.81 ± 1.2	< 0.001	10.19 ± 2.0	8.41 ± 1.4	7.58 ± 0.7	5.59 ± 0.7	< 0.001
Caffein (mg/day)	37.18 ± 9.0	32.06 ± 18.1	36.81 ± 16.6	45.13 ± 21.5	0.076	42.49 ± 17.3	48.48 ± 18.5	34.27 ± 20.0	48.00 ± 8.0	0.164
Alcohol	0.05 ± 0.2	0	0.05 ± 0.2	0.01 ± 0.1	0.212	0.02 ± 0.1	0.01 ± 0.1	0.01 ± 0.1	0	0.294

E-DII, energy-adjusted dietary inflammatory index; SFA, saturated fatty acid; MUFA, monounsaturated fatty acid; PUFA, polyunsaturated fatty acid; BMI, body mass index; Q, quartile

Q1 refers to score indicating the most anti-inflammatory diet, and Q4 refers to scores indicating the most pro-inflammatory diet

[a] ANOVA test for continuous data

**p < 0.05 were considered as statistically significant.

Table S2. Distribution of food group intakes according to quartile of the energy-adjusted dietary inflammatory index (E-DII) score and BMI categories.

Food groups (g/day)	BMI < 25 kg/m^2 n = 157 Quartiles of E-DII					BMI ≥ 25 kg/m^2 n = 156 Quartiles of E-DII				
	Q1	Q2	Q3	Q4	*p*-Value [a]	Q1	Q2	Q3	Q4	*p*-Value [a]
Cereals/grain products	446.64 ± 188.9	477.88 ± 175.9	523.98 ± 192.0	598.26 ± 193.5	< 0.001	463.52 ± 196.5	504.10 ± 188.7	568.40 ± 125.3	610.24 ± 211.9	< 0.001
Meat/meat products	94.18 ± 44.4	76.98 ± 29.4	86.31 ± 33.3	86.04 ± 40.7	0.231	97.77 ± 30.8	107.19 ± 53.4	136.24 ± 54.7	150.46 ± 58.6	< 0.001
Vegetables	294.75 ± 81.9	190.53 ± 103.5	207.83 ± 42.1	104.53 ± 98.2	< 0.001	258.90 ± 44.9	207.86 ± 162.3	115.08 ± 97.5	84.84 ± 68.6	< 0.001
Fruits	319.09 ± 162.0	251.56 ± 187.48	174.33 ± 75.4	100.74 ± 66.5	< 0.001	294.61 ± 219.2	245.69 ± 102.1	184.08 ± 268.4	121.85 ± 130.8	< 0.001
Sugar-sweetened beverages	135.13 ± 69.6	163.18 ± 76.7	192.83 ± 77.5	210.33 ± 82.2	< 0.001	180.63 ± 89.5	226.90 ± 301.2	321.43 ± 274.9	376.90 ± 326.2	0.035**
Fish and seafood	88.08 ± 11.4	77.90 ± 15.7	82.82 ± 17.6	82.81 ± 17.2	0.417	94.82 ± 23.9	91.30 ± 12.5	90.72 ± 19.0	87.32 ± 10.8	0.827
Milk and dairy products	13.74 ± 51.5	19.89 ± 54.8	2.75 ± 17.2	16.40 ± 34.9	0.340	44.97 ± 83.9	37.89 ± 90.4	35.74 ± 47.5	33.19 ± 66.6	0.935
Nuts and legumes	17.49 ± 35.6	13.97 ± 26.6	16.81 ± 22.3	8.86 ± 15.3	0.568	27.43 ± 35.6	24.74 ± 64.5	9.89 ± 17.4	5.27 ± 7.9	0.018**
Condiments	18.91 ± 22.8	26.70 ± 26.8	31.18 ± 11.9	35.74 ± 11.2	< 0.001	10.12 ± 11.9	17.69 ± 17.1	22.77 ± 21.4	28.43 ± 18.8	< 0.001
Confectionary	69.96 ± 28.7	61.18 ± 39.7	53.11 ± 26.4	62.61 ± 51.6	0.202	35.81 ± 29.8	52.49 ± 39.9	66.60 ± 35.9	72.97 ± 30.0	0.017**
Fast foods	6.76 ± 19.0	14.59 ± 17.2	28.02 ± 43.6	28.68 ± 38.8	0.007**	6.99 ± 16.0	12.50 ± 14.6	22.77 ± 24.3	31.82 ± 39.9	< 0.001

E-DII, energy-adjusted dietary inflammatory index; BMI, body mass index; Q, quartile

Q1 refers to score indicating the most anti-inflammatory diet, and Q4 refers to scores indicating the most pro-inflammatory diet

[a] ANOVA test for continuous data

**p < 0.05 were considered as statistically significant.
